# Infants Generalize Representations of Statistically Segmented Words

**DOI:** 10.3389/fpsyg.2012.00447

**Published:** 2012-10-29

**Authors:** Katharine Graf Estes

**Affiliations:** ^1^Department of Psychology, University of California DavisDavis, CA, USA

**Keywords:** statistical learning, word segmentation, language acquisition, word learning, speech perception, generalization

## Abstract

The acoustic variation in language presents learners with a substantial challenge. To learn by tracking statistical regularities in speech, infants must recognize words across tokens that differ based on characteristics such as the speaker’s voice, affect, or the sentence context. Previous statistical learning studies have not investigated how these types of non-phonemic surface form variation affect learning. The present experiments used tasks tailored to two distinct developmental levels to investigate the robustness of statistical learning to variation. Experiment 1 examined statistical word segmentation in 11-month-olds and found that infants can recognize statistically segmented words across a change in the speaker’s voice from segmentation to testing. The direction of infants’ preferences suggests that recognizing words across a voice change is more difficult than recognizing them in a consistent voice. Experiment 2 tested whether 17-month-olds can generalize the output of statistical learning across variation to support word learning. The infants were successful in their generalization; they associated referents with statistically defined words despite a change in voice from segmentation to label learning. Infants’ learning patterns also indicate that they formed representations of across word syllable sequences during segmentation. Thus, low probability sequences can act as object labels in some conditions. The findings of these experiments suggest that the units that emerge during statistical learning are not perceptually constrained, but rather are robust to naturalistic acoustic variation.

## Introduction

Very early in development, infants perform impressive feats of learning. Investigations of statistical learning have revealed that infants rapidly detect distributional patterns that are present in novel visual and auditory input (e.g., Saffran et al., 1996, 1999; Kirkham et al., 2002, 2007). Within the domain of language, statistical learning is hypothesized to support the acquisition of many levels of linguistic structure, from sounds (e.g., Maye et al., 2002), to words (e.g., Saffran et al., 1996; Graf Estes et al., 2007), to syntax (e.g., Gomez, 2002; Mintz, 2003; see recent reviews by Romberg and Saffran, 2010; Thiessen et al., in press). The experimental evidence leaves little doubt that infants can detect statistical regularities in linguistic input. However, there is much less evidence regarding the degree to which the mechanisms at work in statistical learning experiments can contribute to development. A crucial question remains: is statistical learning *useful* for language acquisition? It is not yet clear whether the representations that emerge from statistical learning possess the characteristics that are necessary to support language acquisition and processing.

Effective language processing requires that representations of words be appropriately abstract. They must not be limited to the specific perceptual details of a given word token. Rather, phonological representations must be flexible and generalizable across variation in how words sound because each token of a word varies based on characteristics such as the speaker’s vocal tract, articulatory patterns, accent, speaking rate, and speaking register, as well as the surrounding words and prosodic patterns of the utterance (e.g., Peterson and Barney, 1952; see also reviews in K. Johnson, 2008; Luce and McLennan, 2008; Nygaard, 2008). This presents a significant challenge to young language learners who do not yet know which acoustic variations signify meaningful differences between words and which do not. The ubiquitous variation in speech also presents a challenge to statistical learning accounts of language acquisition. Recognizing sound sequences across acoustically distinct tokens is necessary in statistical learning. In order to track distributional information, infants must detect when the same phonemes, syllables, and/or words occur in different utterances. In addition, to take advantage of prior statistical learning, infants must identify previously discovered patterns when they occur in different contexts or voices. Generalizing from statistical learning experience is crucial for infants to build future learning from prior learning.

The present experiments investigate infants’ ability to generalize statistical learning experience by examining statistical word segmentation, the process of using statistical cues to detect words in fluent speech. Infants were given the opportunity to segment words from a continuous speech stream based on patterns of syllable co-occurrences (i.e., transitional probabilities). Testing then probed whether representations of statistically segmented words are robust to the challenges presented by acoustic variation.

For adults, word recognition is quite resilient to variations in the surface form characteristics of words, which are acoustic variations that do not signal differences in word meaning, such as voice, affect, and accent. These characteristics are encoded during speech processing, but adults adapt quickly (reviewed in Johnson, 2008; Luce and McLennan, 2008; Nygaard, 2008). However, recognizing words across surface form changes is difficult for infants. Houston and Jusczyk (2000) found that 7.5-month-olds failed to recognize words embedded in native language (English) passages of continuous speech when the voice during familiarization differed in gender from the voice used in testing. Infants successfully detected the words when the gender of the voice was consistent. Singh et al. (2004) reported that 7.5-month-olds failed to recognize words across variation in the speaker’s affect. For example, infants familiarized with words in a happy voice recognized them when they were embedded in passages produced with happy affect, but not with neutral affect. Singh et al. (2008b) found that changes in voice pitch (but not amplitude) had a similar effect. Bortfeld and Morgan (2010) also reported that 7.5-month-olds have difficulty detecting familiarized words in passages when stress characteristics of the words change (i.e., from emphatic to non-emphatic stress, or vice versa) between familiarization and testing. These studies indicate that early native language word recognition is inhibited by many acoustic variations that are irrelevant to lexical identity, variations that would have little effect on mature speech processing.

Several factors influence infants’ ability to generalize lexical representations across surface form variation. One important factor is the type of experience that infants have had with the words. When infants hear variable word tokens during familiarization, even 7.5-month-olds can detect those words in sentences across surface form changes (Houston, 2000; Singh, 2008). Infants’ prior word knowledge also matters. Singh et al. (2008a) showed that young infants recognize words across changes in voice pitch if the words are highly familiar items like *Mommy* and *Daddy*, but not when words are unfamiliar. There are also developmental changes in the resilience of infant word recognition, so that by 10.5 months of age, infants can recognize words across changes in voice, pitch, and affective styles (Houston and Jusczyk, 2000; Singh et al., 2004, 2008b; see also Schmale and Seidl, 2009; Schmale et al., 2010 for effects of accent on infant word recognition). This increased sophistication is likely tied to infants’ accumulation of varied experiences and increased word knowledge. By the end of the first year of life, infants’ ability to recognize native language words expands; they are no longer misled by many surface form variations. This expansion occurs at around the same age that infants’ speech perception narrows to focus on sound categories that are meaningfully distinct in their native language (e.g., Werker and Tees, 1984; Werker and Lalonde, 1988; reviewed in Saffran et al., 2006).

The studies investigating how infants cope with surface form variation during word recognition highlight a crucial process in language acquisition. To recognize words, infants must develop lexical representations that are abstract and flexible. They must attend to differences that make meaningful distinctions between words and generalize across irrelevant surface form variants. However, studies of the mechanisms that underlie word segmentation, such as statistical learning, have not explored the effects of acoustic variation.

Many statistical learning experiments present listeners with highly controlled speech streams, produced in a consistent voice throughout learning and testing (e.g., Aslin et al., 1998; Johnson and Jusczyk, 2001; Thiessen and Saffran, 2003). Learners are not required to perform the acoustic generalizations that are necessary in natural language processing, so it remains unclear whether infants can generalize statistical learning experience. During statistical word segmentation, infants may form rigid representations that are constrained by the perceptual details of the input. This would suggest that statistical learning tasks measure lab-based mechanisms with little potential for the flexibility that language acquisition requires. Alternatively, infants may form representations of statistically defined words that are robust to acoustic variation. By the age that infants readily recognize native language words across changes in surface form (Houston and Jusczyk, 2000; Singh et al., 2004, 2008b), they may also readily recognize newly segmented words across variation. This finding would support the hypothesis that statistical learning can meet naturalistic language processing challenges. If statistical learning is a viable contributor to language acquisition, learners must form generalizable representations of the units they extract.

The present experiments investigate whether infants can generalize the representations that emerge during statistical learning. Across two experiments, infants heard the same statistical word segmentation experience. However, two different age groups were tested, 11- and 17-month-olds, with distinct methods designed to tap key learning processes occurring at each age.

During the first year of life, infants’ ability to detect words in fluent speech develops substantially (e.g., Jusczyk, 1997). Therefore, Experiment 1 examined generalization in a traditional statistical word segmentation task with 11-month-olds. During the segmentation phase, infants listened to an artificial language in which the only reliable word boundary cue was transitional probability information. Transitional probability is a conditional probability statistic that indicates the predictive association between two elements. It is calculated based on the frequency of occurrence of a sequence *XY* divided by the frequency of *X* alone. When the sequence *XY* occurs reliably (as occurs within words), transitional probably is high, but when the sequence is inconsistent (as occurs across word boundaries), transitional probability is low. The artificial language exaggerated the pattern that occurs in natural languages (Harris, 1955): within words, syllable co-occurred consistently (i.e., perfect transitional probability); across word boundaries, transitional probability was substantially lower. Similar to prior statistical learning experiments (e.g., Saffran et al., 1996; Aslin et al., 1998), to demonstrate successful learning, infants must discriminate between the high probability words from the language and the low probability sequences that crossed word boundaries, termed part-words. In the present experiment, infants were required to generalize beyond the perceptual details of the segmentation speech stream. Specifically, the infants must segment the words from a language produced by a female voice, then recognize the words in a male voice during testing. If infants form generalizable representations, they should recognize the statistically defined words when they are presented in a new, acoustically distinct voice.

During the second year, a major developmental task is for infants to associate the sounds of words with their meanings. Therefore Experiment 2 tested 17-month-olds in a statistical word segmentation task integrated with a word learning task. Infants listened to an artificial language segmentation phase followed by a label-object association task. Integrating word segmentation and word learning presents an opportunity to investigate the nature of the representations that infants form during statistical learning. It is possible to examine how infants use the units that they discover. In a previous study employing this method, Graf Estes et al. (2007) found that infants took advantage of prior statistical learning to associate novel objects with their labels. They readily learned high probability words from the artificial language as object labels, but failed to learn low probability part-words as labels. Graf Estes et al. proposed that during statistical learning infants form candidate words that are ready to be associated with meanings.

In Graf Estes et al.’s (2007) study, the same female voice presented the segmentation phase and the object labels. Thus, it is not clear whether infants’ representations of candidate words possess the flexibility necessary to facilitate word learning when surface form characteristics change. To investigate this process, the segmentation phase in Experiment 2 was presented in a female voice, but the labels were presented in a male voice. For one group of infants, the object labels were words from the language that the infants had prior opportunity to segment. Alternatively, the labels were part-word sequences that spanned word boundaries in the language (Experiment 2A). If statistical segmentation yields generalizable word like representations, these units should subsequently be available to support lexical functions, such as labeling objects. A follow-up experiment also tested infants’ learning of the labels with no segmentation phase and therefore no prior exposure to the sequences (Experiment 2B).

The variation inherent to speech presents a substantial challenge to learning that learning theories must explain. The present experiments explore whether infants’ statistical learning can meet this challenge. They present two approaches to investigating the abstractness of statistical learning. Experiment 1 tested whether during word segmentation, infants form generalizable acoustic representations of the units they detect. Experiment 2 addressed the underlying representations of statistically defined words, examining whether infants extract and store flexible word like representations that support learning of new object labels.

## Experiment 1

Experiment 1 examined whether infants form generalizable representations during statistical word segmentation. In the *inconsistent voice condition*, infants listened to an artificial language produced in a female voice during the segmentation phase of the task. During the test phase, a male voice produced the test items. In the *consistent voice* *condition*, the segmentation phase was identical to the inconsistent voice condition. However, the test items were produced by the same female voice as infants heard during segmentation. The purpose of the consistent voice condition was to establish 11-month-olds’ learning pattern for these stimuli when the voice is consistent from segmentation to testing. If infants learn the structure of the artificial language, they should show a difference in listening time between the low transitional probability part-words versus the high transitional probability words. In statistical learning experiments, infants typically display a novelty preference for the part-words (e.g., Saffran et al., 1996; Aslin et al., 1998).

### Materials and methods

#### Participants

Fifty-six infants were randomly assigned to the consistent and inconsistent voice conditions (28 infants per condition; 35 males and 21 females). The average age was 11.1 months (SD = 0.23; range 10.2–11.5 months). The infants were born full term and were free of vision and hearing problems, according to parental report. The infants all came from homes in which English was the predominant language spoken. Based on parental interviews, 15 of the infants had some exposure to a second language, 20 h per week or less (*n* = 5 in the consistent voice condition, *n* = 10 in the inconsistent voice condition). The results of the experiment are unchanged if the infants with second language exposure are excluded from the analyses. In the consistent voice condition, two additional infants were identified as outliers based on listening time differences to words versus part-words that were over 2.5 SD from the mean. These infants were excluded from analyses. An additional 17 infants were excluded because of fussiness (*n* = 8 in the consistent voice and *n* = 9 in the inconsistent voice conditions). The University of California, Davis Institutional Review Board approved the research protocol for Experiments 1 and 2. The parents of our participants gave informed consent.

#### Stimuli

The artificial language used in the segmentation phase was originally developed by Graf Estes et al. (2007). To control for infants’ arbitrary listening preferences, there were two counterbalanced versions of the artificial language. The words in Language 1 were *timay, dobu, gapi*, and *moku;* the words in Language 2 were *pimo, kuga, buti*, and *maydo*. As shown in Table 1, the counterbalancing resulted in syllable sequences that acted as word test items in Language 1 and part-word test items in Language 2, and vice versa. The artificial language was recorded using a method that approximates the actions of a speech synthesizer. A female speaker recorded 3-syllable sequences, of which the medial syllables were excised and spliced to form the final speech stream (i.e., the recorded sequences *timaydo*, *maydobu*, *dobuga* were spliced to form the sequence *maydobu*). Recording 3-syllable sequences allowed for natural coarticulation of each syllable. Splicing the medial syllables to form a fluent sequence reduced the chance for the speaker to inadvertently introduce additional word boundary indicators. The speech stream contained no pauses or other reliable acoustic cues to word boundaries. The only reliable word boundary cues were the transitional probabilities of syllable sequences. The within-word transitional probabilities were 1.0 (i.e., the syllables within each word always occurred together) and the across word probabilities ranged from 0 to 0.5. The duration of each speech stream was 5.5 min.

**Table 1:** **Word and part-word test items for Experiments 1 and 2**.

	Words	Part-words
Language 1	*timay, dobu*	*pimo, kuga*
Language 2	*pimo, kuga*	*timay, dobu*

The artificial language was designed to equate the frequency of the word and part-word test items, but maintain the difference in their transitional probabilities. Using this design, it is possible to determine whether infants discriminate words from sequences that occur with equal frequency in the artificial language, but differ in their internal statistical structure (Aslin et al., 1998). To balance the frequency of the test items, the language contained two high frequency words that occurred 180 times in the speech stream (Language 1: *gapi* and *moku*; Language 2: *buti* and *maydo*) and two low frequency words that occurred 90 times (Language 1: *timay* and *dobu*, Language 2: *pimo* and *kuga*). This design yielded two part-words that occurred 90 times in the speech stream, occurring at the conjunction of the two high frequency words. For example, in Language 1, *gapi* preceded *moku* 90 times. Therefore, the part-word sequence *pimo* occurred the same number of times as the low frequency words (e.g., *timay*). The test items were the low frequency words and the part-words formed from the high frequency words (see Table 1). All occurred 90 times during the segmentation phase. However, the words had perfect transitional probability (transitional probability = 1.0) and the part-word sequences contained a dip in transitional probability between syllables (transitional probability = 0.5).

In the consistent voice condition, the same female speaker recorded the artificial language and the test items. In the inconsistent voice condition, a male speaker recorded the test items. The average fundamental frequency (*F*0, a measure of pitch) of the male voice test items was 121 Hz, which was substantially lower than the fundamental frequency of the artificial language (224 Hz) and the female voice test items (234 Hz). The test items were recorded in citation form, with a monotone speaking style in order to maintain similarity with the speech from the segmentation phase. Repetitions of the test items were separated by 750 ms of silence. All sounds were played at a level approximating conversational speech, around 65 dB.

#### Procedure

During the segmentation phase, each infant and his or her parent were allowed to move around a sound attenuated booth while playing quietly. The parent was instructed not to refer to the artificial language and to remain as quiet as possible. Following the segmentation phase, the parent and child were moved to a second sound attenuated booth. In the test booth, a television at the front of the room displayed visual animations and attention-getting stimuli and broadcast the sound sequences. The infant sat on the parent’s lap approximately 1 m from the screen. A camera mounted below the television screen enabled the observer, located outside the booth, to monitor looking behavior. When the parent and child entered the test booth, the parent heard a brief reminder about the instructions for the test phase of the experiment. Because of this delay, the infant received a 30 s refamiliarization with the artificial language before testing. The refamiliarization was paired with a silent cartoon clip to maintain the infant’s interest.

The program Habit X (Cohen et al., 2004) was used to present infants with the test items in an auditory preference procedure. As a protection against bias, the experimenter was blind to the identity of the materials being presented, and the parent listened to masking music over headphones. Test trials immediately followed the refamiliarization. Each trial consisted of repetitions of a word test item or a part-word test item. There were 16 test trials. The four test items (two words, two part-words) were presented in four randomized blocks.

To measure infants’ listening time to the auditory test items, all items were paired with a visual animation of an orange oval turning in a circle on the screen. The presentation of the test trials was contingent on the infant’s looking at the visual animation. Using a button press, the experimenter indicated how long the infant’s attention remained fixated on the audio-visual item. The test item repeated until the infant looked away for 1 s or after a maximum listening time of 20 s. To regain the infant’s interest, a cartoon played between trials.

The program Habit X tallied listening time to each test item. The dependent measure was based on listening time (indicated by attention to the audio-visual stimuli) to the word and part-word test items. The measure of listening time used here is similar to the central fixation procedure used by Shi and Werker (2001; Shi et al., 2006) and the visual fixation-based auditory preference procedure used by Cooper and Aslin (1990, 1994). It is also similar to the head turn preference procedure frequently used in statistical learning experiments (Saffran et al., 1996; Aslin et al., 1998; Johnson and Jusczyk, 2001).

### Results and discussion

Preliminary analyses revealed that there were no differences in performance based on sex or artificial language version (Language 1 versus Language 2). Therefore, subsequent analyses collapsed across these variables.

**Figure 1:**
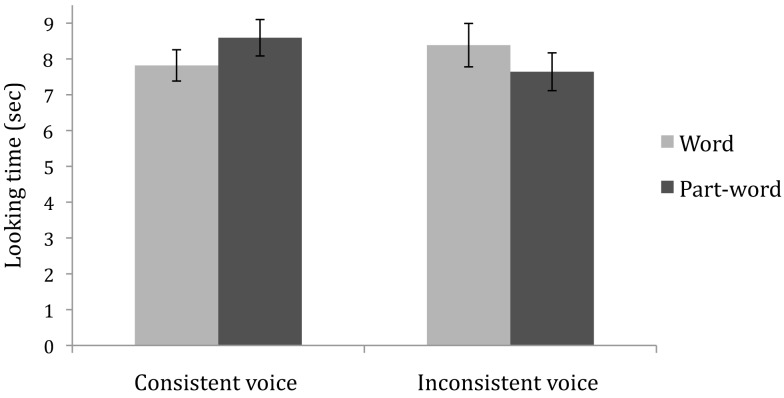
**Mean looking time (in seconds) to word versus part-word test trials**. Error bars represent standard errors.

Infants’ learning was analyzed in a 2 (Condition: consistent voice vs. inconsistent voice; between subjects) × 2 (Trial type: word vs. part-word; within subjects) mixed ANOVA. There was no main effect of condition and no main effect of trial type, *F*’s < 1. There was a significant interaction of condition by trial type, *F*(1, 54) = 12.4, *p* = 0.001, $\eta_{p}^{2}=0.19$ To explore the interaction, each condition was analyzed separately with a paired samples *t*-test comparing looking time to word versus part-word test trials. In the consistent voice condition, infants listened significantly longer to the part-words, *t*(27) = 2.56, *p* = 0.016, *d* = 0.31. In the inconsistent voice condition, infants listened significantly longer to the words, *t*(27) = −2.41, *p* = 0.023, *d* = 0.25. Listening time performance is illustrated in Figure 1. In the consistent voice condition, 20 of 28 infants showed the novelty preference for part-words. In the inconsistent voice condition, 17 of 28 infants showed the familiarity preference for words.

In both conditions, infants discriminated the word versus part-word test items, indicating that they learned the structure of the artificial language, and recognized the words from the speech stream. However, the infants showed different directions of preference. The part-word preference in the consistent voice condition follows the pattern of many statistical learning experiments (Saffran et al., 1996; Aslin et al., 1998; Johnson and Jusczyk, 2001; Thiessen et al., 2005; Experiment 2) and is typically interpreted as a novelty preference for the items that were not previously detected in the segmentation phase. The preference for word test items has been demonstrated in some experiments (Saffran, 2001; Thiessen et al., 2005, Experiment 1; Thiessen and Saffran, 2003, Experiment 1). According to Hunter and Ames’s (1988) model of infants’ attentional preferences, infants display novelty preferences when information has been thoroughly processed (see also Houston-Price and Nakai, 2004). Infants are likely to display familiarity preferences when a task is difficult. One characteristic that affects task difficulty is the match between the familiarization stimuli and test items (Hunter and Ames, 1988; Thiessen and Saffran, 2003). When test items are similar to the familiarization stimuli, the task is easier than when the test items differ from familiarization. The novelty preference displayed in the consistent voice condition and the familiarity preference displayed in the inconsistent voice condition suggest that recognizing the words in the familiar voice was easier for infants than recognizing the words in the novel voice.

In Experiment 1, 11-month-olds performed a linguistically relevant generalization across acoustic variation in a statistical learning task. The infants’ representations of the statistically segmented word forms were sufficiently abstract to recognize the words when they were produced in a novel, acoustically distinct voice during testing. This is very close to the age at which infants readily recognize native language words across changes in affect (Singh et al., 2004) and speaker’s voice (Houston and Jusczyk, 2000), 10.5 months. The similar age across experiments highlights the notion that infants are processing speech in a similar way when it is produced in their native language or an artificial language. In addition, our findings are consistent with a recent experiment by Vouloumanos et al. (2012), who found that adults readily identify statistically defined words across a change in voice. For highly experienced adult language processors, performance was not different when recognizing the words in the same voice or a different voice. For infants, the change in direction of preference suggests that generalizing across voices is more difficult than recognizing words when the voice is consistent. Yet the infants’ representations of statistically segmented units are not limited by the perceptual details of their learning experience.

## Experiment 2

Experiment 1 demonstrated that statistically segmented units are robust to surface form variation. Such generalization is necessary for recognizing words and accumulating information about the meanings and uses of words. However, the listening time measure used in Experiment 1, and in many other statistical learning experiments, is limited in what it can reveal about the representations that infants form during statistical learning. Listening preference measures are highly valuable tools. Infants’ discrimination of high and low transitional probability sequences demonstrates that they are powerful learners, able to rapidly detect structure in linguistic input based on limited information. But infants’ discrimination performance alone cannot tell us whether the representations formed during statistical learning are mere sounds, or whether they have any linguistic status (Saffran, 2001). To directly explore the nature of the representations that infants form during statistical learning, it is necessary to design tasks that test how infants apply the output of statistical learning to other linguistic processes. If the output of statistical word segmentation is word like units, infants should be able to use those units to perform the kinds of tasks that real words perform.

To address this issue, Graf Estes et al. (2007) designed a task that integrates statistical word segmentation with word learning. Infants first participated in a segmentation phase during which they heard an artificial language. The segmentation phase was immediately followed by a label-object association task, rather than a listening preference measure. The same (female) voice presented the segmentation phase and labeling task. The label-object association task presented a simplified word learning event (Werker et al., 1998). Infants habituated to two label-object pairs. After habituation, infants’ learning was measured by the duration of their looking time on test trials in which they viewed the original label-object pairs or trials in which the original associations were violated. If infants have learned the labels, they should look longer on the trials in which the learned pairings were violated.

Using this method, Graf Estes et al. found that 17-month-olds readily learned statistically defined words as object labels. However, infants failed to learn labels that were part-words or non-words (novel sequences of syllables from the language). As in Experiment 1, the word and part-word test items occurred with equal frequency during the segmentation phase. Therefore, before they occurred as labels infants heard the words and part-words equally often, but the items differed in their internal transitional probabilities. Graf Estes et al. (2007) concluded that transitional probability information was weighted more heavily than frequency information in determining whether a sound sequence was a good potential object label. The findings also indicate that infants can use statistical learning to extract candidate words that are then available to be associated with meanings (for related findings with adults see Mirman et al., 2008 and Endress and Mehler, 2009 for a counterargument).

Experiment 2 used the method designed by Graf Estes et al. (2007) to examine infants’ ability to use the output of statistical learning in a word learning task when infants must generalize across acoustic variability in order to do so. The participants were 17-month-olds because at this age, the process of associating sounds with meanings is a major focus of language acquisition. This age group also allows for a direct comparison with previous experiments examining the connection between statistical word segmentation and word learning (Graf Estes et al., 2007; Hay et al., 2011).

The stimuli in Experiment 2 came from the inconsistent voice condition of Experiment 1. The segmentation phase was presented in a female voice and the label-object associations were presented in a male voice. For half of the infants, the labels were words from the artificial language. For the other half of the infants, the labels were part-words. If infants form generalizable representations of candidate words, Experiment 2 should replicate the findings from Graf Estes et al. (2007) when the voice changes from segmentation to label learning. Statistical word segmentation should support infants’ learning of novel object labels when the labels are newly segmented words, but not when the labels are part-word sequences.

## Experiment 2A

### Materials and methods

#### Participants

Forty-four infants were randomly assigned to the word and part-word label conditions (22 infants per condition; 22 males, 22 females). The average age of the participants was 17.3 months (SD = 0.34; range 16.6–17.8 months). All infants were born full term and had no history of hearing or vision impairments. Based on parental interviews, eight infants had some exposure to a second language, 20 h per week or less (*n* = 5 in the word condition and *n* = 3 in the part-word condition). The results of the experiment are unchanged if infants with second language exposure are excluded from the analyses. Twenty-three additional infants were excluded because of fussiness (*n* = 19), moving out of the video frame (*n* = 3), and experimenter error (*n* = 1). In the part-word condition, one additional infant was identified as an outlier based on a looking time difference to same versus switch test trials that was greater than 2.5 SD from the mean. The infant was excluded from the analyses.

#### Stimuli

##### Word segmentation task

The artificial language was the same as the language used in Experiment 1. It was presented in a female voice. The test items were identical to the word and part-word sequences presented in the inconsistent voice condition (male voice) of Experiment 1.

##### Object labeling task

**Figure 2:**
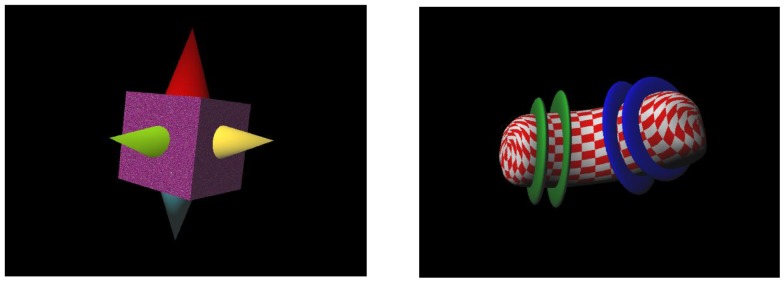
**Novel objects that received labels**.

The novel objects, shown in Figure 2, were two computerized 3-D images designed to be visually complex and discriminable in shape and color. Each object was paired with an object label. For all infants, the labels were presented in a male voice. For half of the infants, the object labels were words from the artificial language (e.g., *timay* in Language 1). For the other half of the infants the object labels were part-words (e.g., *kuga* for Language 1). Because of the artificial language design (see Experiment 1), the word and part-word labels occurred equally frequently during the segmentation phase, but differed in their internal transitional probabilities.

Each infant participated in one of four testing conditions: half of the infants exposed to Language 1 received two word test items, and half received two part-word test items. Half the infants exposed to Language 2 received two word test items, and half received two part-word test items. The test items are shown in Table 1.

#### Procedure

The method for presenting the artificial language in the word segmentation phase was identical to the method described in Experiment 1. The infant listened to the language in a sound attenuated booth and heard a 30 s refamiliarization after being moved to the testing booth. Instead of measuring infants’ discrimination of word and part-word test items, the infants immediately participated in a label-object association task. A version of the Switch task was used to test infants’ learning of label-object pairings (Werker et al., 1998). It is a popular measure of early word learning with low task demands. Although the Switch task lacks the social referential context that is present in interactive word learning tasks, it retains a fundamental component of the word learning process – linking a sound sequence representation with a meaning representation (here, object identity). The measure has been used recently in several studies to investigate factors affecting early word learning (Fennell et al., 2007; Curtin, 2009; Rost and McMurray, 2009).

The program Habit X was used to present the label-object combinations in the Switch task. As a protection against bias, the experimenter was blind to the identity of the materials being presented, and the parent listened to masking music over headphones. The infant started the task with a familiarization trial that allowed the infant to become accustomed to the audio-visual stimuli presentation before the first habituation trial. The infant viewed a rotating gray screen presented on a black background accompanied by repetitions of the syllable “neem.”

During the habituation phase, the infants viewed two label-object combinations. Each label-object combination was presented one at a time, with the order randomized by blocks. The object moved from side to side while its associated label played. Each label repetition was separated by 750 ms of silence. Presentation of the stimulus continued as long as the infant remained fixated on it. Trials terminated when the infant looked away for 1 s, or for a maximum of 20 s. A cartoon played between trials to guide the infant’s attention back to the screen. The habituation criterion was satisfied when the infant’s average looking time on three consecutive trials decreased to 50% of the average looking time on the first three habituation trials.

Test trials began immediately after the infant reached the habituation criterion or viewed a maximum of 25 habituation trials. There were two types of test trials: on *same* test trials, the original label-object associations from habituation were maintained. On *switch* test trials, the label-object pairings were violated (e.g., object 1 was presented with label 2). There were four same and four switch test trials, organized in two counterbalanced testing orders. In both orders, the switch test trials occurred first, which provides infants with the best opportunity to display learning in case infants’ attention wanes throughout testing. These test orders replicate the orders that Graf Estes et al. (2007) used. When the label voice matched the segmentation voice, they found that infants learned the word object labels, but not the part-word labels. Thus, although the test orders give infants the strongest chance to display learning, it is possible for infants to fail to display learning of the labels using test orders in which switch trials are presented first (see also Experiment 2B).

### Results and discussion

Preliminary analyses revealed no significant differences in performance based on sex or language version (Language 1 versus 2). Therefore, subsequent analyses collapsed across these variables.

In the word label condition, infants reached the habituation criterion in a mean of 11.5 trials (SD = 5.8). In the part-word condition, infants reached the habituation criterion in a mean of 11.2 trials (SD = 5.3). There was no significant difference in the number of trials to reach habituation, *t*(42) = 0.163, *p* = 0.872, *d* = 0.05. One infant in the word label group and one in the part-word label group failed to habituate. The results of the analyses are unchanged if these infants are excluded.

**Figure 3:**
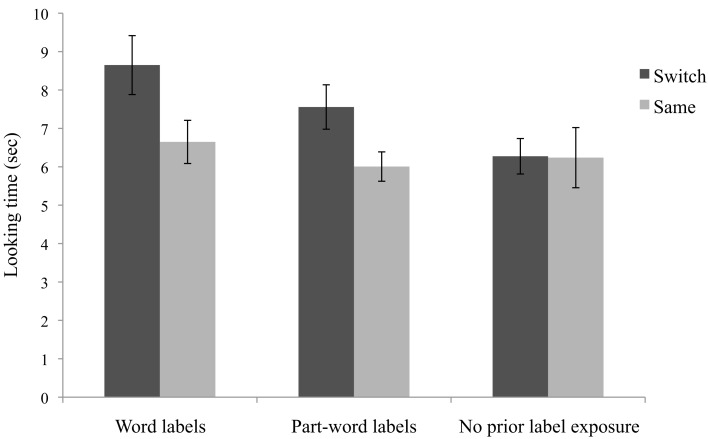
**Mean looking time (in seconds) to word and part-word labels (Experiment 2A), and labels with no prior segmentation phase exposure (Experiment 2B) to the same and switch test trials**. Error bars represent standard errors.

Infants’ learning was analyzed in a 2 (Label condition: word versus part-word; between subjects) × 2 (Trial type: same versus switch; within subjects) mixed ANOVA. There was no main effect of label condition, *F*(1, 42) = 1.64, *p* = 0.207, $\eta_{p}^{2}=0.04$ and no interaction of label condition by trial type, *F *< 1. There was a main effect of trial type, *F*(1, 42) = 13.46, *p* = 0.001, $\eta_{p}^{2}=0.24.$ Follow-up paired samples *t*-tests confirmed that infants in the word label condition showed significantly longer looking on the switch test trials, *t*(21) = 2.47, *p* = 0.022, *d* = 0.64. Infants in the part-word label condition showed the same pattern, *t*(21) = 2.94, *p* = 0.008, *d* = 0.69. Fifteen of 22 infants in the word label condition and 17 of 22 infants in the part-word label condition looked longer on the switch test trials than the same trials. Looking time is illustrated in Figure 3.

**Figure 4:**
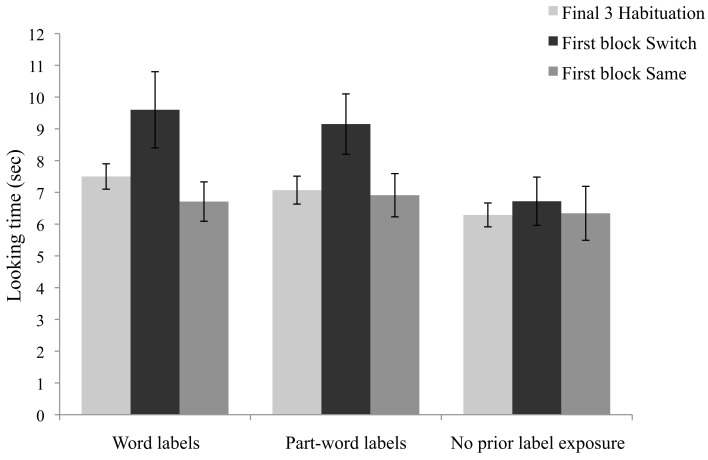
**Mean looking time (in seconds) to word and part-word labels (Experiment 2A), and labels with no prior segmentation phase exposure (Experiment 2B) during the final three habituation trials and the first block of switch and same test trials**. Error bars represent standard errors.

The analyses of the same and switch test trials indicate that infants learned both the word and part-word labels; they detected when the label-object pairings were switched. In a previous experiment using a consistent voice, but the same task and test orders, infants who heard word labels showed significantly longer looking on switch trials, but infants who heard part-word labels did not (Graf Estes et al., 2007). However, it is theoretically possible that the difference in looking time to the same and switch trials occurred here because the test phase began with switch trials and a general decline in attention produced the effect. If the present findings occurred because of declining attention, looking time should also decline from habituation to the first block of test trials. In contrast, if infants learned the label-object pairings during habituation, they should dishabituate to the first switch trials even though the trials occurred later in the experiment. Similar to the analyses above, a 2 (Label condition: word vs. part-word) × 2 (Trial type: habituation versus first two switch trials) ANOVA was performed (Two infants who did not habituate were excluded, but the pattern is the same with these infants included.). Figure 4 shows that across the word and part-word label conditions, infants dishabituated to the switch trials (main effect of trial type: *F*(1, 40) = 8.3, *p* = 0.006; no effect of label condition and no interaction, *p*’s > 0.63). The analysis was repeated for the first block of same test trials versus habituation trials. There were no main effects of trial type or label condition, and no interaction (all *p*’s > 0.35), indicating that looking time between habituation and the first same trials did not differ. This analysis suggests that infants looked longer on switch trials than same trials during testing because they detected that switch test trials differed from the label-object pairings shown during habituation.

## Experiment 2B

Infants displayed evidence of learning both the word and part-word labels in Experiment 2A. This conflicts with previous evidence that infants learn word, but not part-word labels when the same voice presents the segmentation phase and the object labels (Graf Estes et al., 2007). Given the design of Experiment 2A, it is possible that infants learned more effectively from the male test voice than the female test voice that Graf Estes et al. (2007) used. The difference in performance could be unrelated to infants’ statistical segmentation experience. Graf Estes et al. (2007) and Graf Estes and Hurley (in press) also reported that infants failed to learn these labels when they were presented in a monotone or adult-directed female voice with no segmentation phase. Experiment 2B tested whether infants readily learned the labels when they were presented in a male voice, without any prior segmentation experience. Infants only participated in the label-object association task, which should minimize any effects of fatigue during testing, thereby giving infants the best opportunity to display learning. If the male voice labels are simply easy to learn on their own, infants should look longer on the switch test trials than the same trials. However, if exposure to the speech stream before label exposure is important, infants should have difficulty learning the labels.

### Materials and methods

#### Participants

Twenty-two infants participated in this task (10 females, 12 males). The average age was 17.1 months (SD = 0.31; range 16.7–17.9 months). Seven infants had some exposure to a second language. The results of this experiment are unchanged with these participants excluded. An additional seven infants were excluded because of fussiness (*n* = 5), moving out of the camera view (*n* = 1), or equipment or experimenter error (*n* = 1).

#### Stimuli and procedure

The stimuli and procedure were identical to Experiment 2A, except that infants did not participate in the segmentation phase. They went directly to the test booth and participated in the label-object association task. Infants were randomly assigned to hear the labels *timay* and *dobu* or *gapi* and *moku*.

### Results and discussion

Preliminary analyses revealed no significant differences in performance based on sex or labels versions (*timay* and *dobu* vs. *gapi* and *moku*). Therefore, subsequent analyses collapsed across these variables.

Infants reached the habituation criterion in a mean of 10.2 trials (SD = 4.5). All infants met the habituation criterion. A paired samples *t*-test revealed that there was no difference in looking time on same versus switch test trials, *t*(21) = 0.051, *p* = 0.960, *d* = 0.01. Thirteen of 22 infants showed a switch test trial preference. There is no evidence that infants learned the labels in the absence of the opportunity to segment them from fluent speech before they occurred as labels. They failed to display learning even though conditions were designed to minimize fatigue effects.

In contrast to Experiment 2A, Figure 4 shows that infants in Experiment 2B did not look longer during the first block of switch trials compared to the final habituation trials. There was also no difference in looking time between the first block of same trials and the final habituation trials (*p*’s > 0.61). This result further supports the argument that infants’ differential attention to same and switch test trials for the word and part-word labels was not merely due to the test orders combined with a general decline in attention throughout testing.

Across Experiments 2A and 2B, infants learned the statistically defined words and part-words as labels, but failed to learn the same labels in the absence of prior exposure. Infants transferred statistical segmentation experience to support object label learning when it required generalizing beyond the acoustic characteristics of their input. The output of statistical learning is not bound by the perceptual details of the original familiarization stimuli. Rather, infants can perform this naturalistic generalization in service of a real language acquisition task, associating the sounds of words with their referents.

The results also show that infants form and store representations of sequences that are not word units, but rather occur across word boundaries. Although the part-word test items had low transitional probability relative to the words, several characteristics may have facilitated their use in the label learning task. Infants had ample opportunity to hear the part-words before they appeared as object labels; they occurred in segmentation phase 90 times across 5.5 min. In addition, the transitional probability of the part-word labels was 0.5, whereas other word boundary probabilities ranged from 0 to 0.26. These lower transitional probability sequences may have produced clearer word boundaries than the across word sequences that occurred as labels. In natural languages, word-internal transitional probabilities are rarely perfect. Some real words may contain probabilities closer to the 0.5 value of the part-words than the 1.0 value of the words examined here. Based on their frequency and transitional probability patterns, the part-words may have formed relatively coherent sequences, available to support label learning. However, this rationale and the present results conflict with Graf Estes et al.’s (2007) findings when the voice presenting the object labels matched the voice during segmentation. The General Discussion proposes an explanation for the divergent results. Nonetheless, the present findings demonstrate that infants form and retain representation of the sequences that cross word boundaries in addition to representations of the coherent, high transitional probability word units.

## General Discussion

In this series of experiments, infants participated in tasks tailored to investigate two different stages of language acquisition. Experiment 1 examined statistical word segmentation in 11-month-olds and found that infants can recognize statistically segmented words across variation in a speaker’s voice. Experiment 2 examined whether 17-month-olds can generalize the output of statistical word segmentation across variation to support object label learning. The infants were successful; they associated referents with statistically defined words, as well as with frequently occurring sequences that spanned word boundaries in the speech stream. Across Experiments 1 and 2, infants heard the same stimuli, but testing tapped different language acquisition processes. Each experiment has an independent contribution to understanding the representations infants form during statistical learning. In addition, combining the methods of testing word segmentation and label learning following segmentation has revealed characteristics of learning that would not have been apparent from either experiment alone (see also Pelucchi et al., 2009; Hay et al., 2011).

In Experiment 1, infants’ discrimination performance showed that they could recognize the statistically segmented words across a change in voice. Similar to many previous statistical word segmentation experiments, infants presented with a consistent voice attended longer to novel part-words than to words (Saffran et al., 1996; Aslin et al., 1998; Johnson and Jusczyk, 2001; Thiessen et al., 2005; Experiment 2). In contrast, infants who heard an inconsistent voice across segmentation and testing showed a familiarity preference for the words. Models of infants’ attentional preferences explain that when a task is relatively easy, or infants have become highly familiar with the training stimuli, novel stimuli elicit greater attention than familiar stimuli. When a task is difficult, there is a greater likelihood that infants will demonstrate a familiarity preference for patterns that are consistent with their training stimuli (Hunter and Ames, 1988). A mismatch between familiarization and test (such as the change in voice in the inconsistent voice condition) is one characteristic that can make a task difficult. Thus, a conclusion from the segmentation task in Experiment 1 is that infants can generalize across statistical segmentation experience, but it is more difficult than recognizing words when the voice is consistent. The label learning measure in Experiment 2 did not reveal this difference in the ease of processing.

Around 11 months of age, infants can recognize native language words across variation in characteristics such as affect, pitch, and voice (Houston and Jusczyk, 2000, 2003; Singh et al., 2004, 2008b). Thus, in Experiment 1, infants showed flexibility in word recognition in statistical learning at around the same age as in their native language. It is not yet clear whether the full developmental trajectory of word recognition across variation is similar in native language word segmentation and statistical word segmentation of artificial languages. It remains to be tested whether younger infants (e.g., 7.5-month-olds) have difficulty recognizing statistically segmented words across variation, as they do for native language words (Houston and Jusczyk, 2000; Singh et al., 2004, 2008a,b; Bortfeld and Morgan, 2010). In addition, future experiments will be necessary to explore the range of flexibility of infants’ representations of statistically segmented words. Vouloumanos et al. (2012) found that adults’ representations are abstract, but within limits. In a statistical learning task, adults recognized words across a change in the speaker’s voice and across some types of distortion. While adult native language word recognition withstands many forms of unnatural variation, such as distortion (Remez et al., 1981; Pisoni, 1996; Saberi and Perrott, 1999), it greatly disrupts infant word recognition (Zangl and Mills, 2007). The effects of unnatural variation on recognizing segmented words may be stronger than the effects of natural variation because infants lack experience with experimentally manipulated unnatural variations (e.g., time reversals or low-pass filtering). By 11 months of age, infants may succeed in recognizing statistically segmented words across the change in voice because native language experience leads them to expect that the same word can sound different depending on who says it.

Experiment 2 combined statistical word segmentation with a label learning task in order to capture a more nuanced picture of statistical learning than the segmentation task alone can provide. This integration yields an understanding of the linguistic status of the representations that infants form by showing how the output of statistical learning can be used to support word learning. In this case, it revealed an unexpected pattern. In contrast to previous findings (Graf Estes et al., 2007), 17-month-olds learned low transitional probability part-word sequences as object labels in addition to statistically coherent, high transitional probability words. Infants’ learning of the part-word labels suggests that they develop and store representations of syllable sequences that cross word boundaries in fluent speech in addition to the sequences that form words.

It is not yet clear why part-word sequences support label learning when infants must generalize their statistical segmentation experience across voices, but not when the voice is consistent throughout segmentation and label learning. One possible explanation is motivated by models of word segmentation and memory (see Thiessen et al., in press, for a more thorough discussion of the integration of memory and statistical learning models). In Perruchet and Vinter’s (1998) Parser model of word segmentation, one process that contributes to learners’ extraction of word units is interference. In Parser, sequences, or chunks, that occur together frequently build up activation. The reliability of a chunk also contributes to its strength of activation. Chunks that occur frequently, but unreliably (like part-words) will not emerge as units because of interference from learning the reliably occurring, high probability units (words). Part-words consist of syllables that belong to the words, so knowledge of the words inhibits learners from segmenting out the part-word sequences (see also Giroux and Rey, 2009). This helps to frame the prior finding that infants learn word labels, but not part-word labels (Graf Estes et al., 2007).

To consider why the change in voice affects label learning, one must also consider memory models that posit that each experience with a word affects its stored lexical representation. In episodic memory models, each exemplar (e.g., each token of a word) is stored as a memory trace and exemplars accumulate over time. When a retrieval cue is presented and the stored exemplars overlap greatly with it (e.g., a word is repeatedly produced in a consistent voice), there is a stronger activation than when the retrieval cue is dissimilar from previous experience (e.g., a word produced in a new voice; Hintzman, 1986; Goldinger, 1996, 1998).

Integrating the episodic memory and word segmentation models suggests the following hypothesis. When the voice is consistent from segmentation to labeling, infants activate detailed representations of the segmentation speech stream because of the high overlap between the retrieval cue (i.e., the label) and prior experience. Infants’ representations of the highly reliable and frequent words are strong; these units can act as object labels. The part-words, although frequent, conflict with the word representations and are therefore not stored as units available for further processing. However, when the voice changes from segmentation to labeling, the mismatch means that activation of prior learning is weaker. Building from the role of interference in Parser, the reduced activation caused by the change in voice could free infants from the inhibition caused by the conflicting representations of the words and part-words. This could then allow infants to use their experience hearing other frequently occurring syllable sequences, like part-words, to promote label learning. This hypothesis leads to the prediction that other conditions that produce weak activation of statistical learning, such as introducing a delay between segmentation and labeling, should reveal stored representations of part-words.

Further consideration of word segmentation models provides additional context for the findings from Experiment 2 and offers new predictions. Clustering and bracketing models present two broad categories of word segmentation strategies that have been explored (Goodsitt et al., 1993; Brent, 1999). Clustering (or chunking) models share the concept that tracking probabilistic information leads learners to extract sequences that occur reliably, yielding statistically coherent word like units (see various instantiations by Perruchet and Vinter, 1998; Swingley, 2005; Giroux and Rey, 2009; Frank et al., 2010). In contrast, bracketing (or boundary-finding) models propose that learners track the relations between elements and infer boundaries between them at points of low probability (e.g., Elman, 1990; Cairns et al., 1997; Christiansen et al., 1998). Learners do not extract cohesive units, but detect areas of low predictability. Evidence that infants readily associate meanings with statistically defined words supports clustering accounts. It suggests that infants extract and store candidate words that are available to feed other linguistic processes.

Clustering models also shed light on why the part-words acted as good object labels. Giroux and Rey (2009) explained that according to clustering models, increased experience with a speech stream should lead to stronger differentiation of items that are and are not words because learning about words should interfere with representations of other frequently occurring sequences. With sufficient experience, words will become the units that are available in memory, not part-word sequences, or sublexical sequences (i.e., syllable pairs within trisyllabic words). Accordingly, they found that after a brief exposure to an artificial language, adults did not differ in their ability to distinguish words and sublexical sequences from part-words. However, after a long exposure, participants identified words more accurately than sublexical units. In contrast, bracketing models predict that increased duration of exposure should not produce stronger differentiation of words and sublexical units because the exposure to and representation strength of words and sublexical units are tightly linked.

Giroux and Rey’s (2009) account raises the possibility that infants in Experiment 2 were still learning about the frequency and reliability of the words in the language. The learning was not sufficiently complete to produce full inhibition of the part-word sequences, at least not when the sequences changed in voice from segmentation to labeling, thereby reducing interference from the word sequences. With greater exposure, the clustering account suggests that infants should show stronger differentiation between word and part-word labels, as well as word and sublexical sequences. Bracketing models would not predict this change.

There is an apparent contrast between infants’ performance in the segmentation task alone (Experiment 1) and in the segmentation task followed by the label learning task (Experiment 2). In the segmentation task, infants differentiated the word and part-word test items, but in the label learning task they did not. It seems unlikely that the age difference across experiments, 11 months versus 17 months, produced the contrasting patterns of performance. Previous studies suggest that children do not lose the ability to perform statistical word segmentation (Saffran et al., 1997; Graf Estes et al., 2007). Rather, the different patterns of learning across experiments reveal that while infants can generalize representations of statistically segmented words, generalization depends on context. Differences in the demands and goals of each task may have encouraged infants to interpret the same stimuli in different ways. The auditory preference task from Experiment 1 is well-suited to measuring infants’ ability to discriminate sound sequences. It presents a within subjects comparison of attention to each test trial type. Hearing the test items in close succession may promote infants’ attention to the differences between them. The auditory preference task revealed a rapid learning and generalization capability, evidenced by infants’ differentiation of items with high and low transitional probability. However, the preference task was not equipped to explore whether the items that infants perceive to be different also differ in their linguistic status (but see Saffran, 2001). Integrating the segmentation and label learning tasks can show whether infants form representations during statistical learning that feed forward to support label learning. However, the design of the label learning task is not well-suited to a direct comparison of the ease or strength of learning because infants hear only one label type (words or part-words). Infants cannot compare the high and low probability test items as they can in the auditory preference task. In addition, the Switch task does not typically indicate precise differences in the strength learning. Infants either show a significant difference in attention to same versus switch test trials or they do not. Thus, it is possible that words and part-words do not serve as equally good object labels, but more sensitive methods (e.g., Yoshida et al., 2009) will be necessary to reveal the difference. This possibility is currently being tested.

The present experiments highlight the importance of using multiple methodologies to investigate a construct. Experiments 1 and 2 examined two interrelated aspects of statistical learning: statistical word segmentation and the representational status of statistically segmented sequences. The combination of findings from these experiments show that generalization in statistical learning is affected by the demands of the problem that infants must solve. The experiments also illustrate limitations of the methods used in each experiment. The auditory preference measure yielded two different statistically significant directions of preference. Although there are precedents for both novelty and familiarity preferences in statistical learning tasks, making the same conclusions (i.e., successful learning) from opposite results can present interpretational challenges. In addition, as discussed above, auditory preference tasks can reveal that infants successfully discriminate sound sequences, but cannot specify the nature of those representations. Integrating statistical word segmentation and word learning, as in Experiment 2, takes a significant step toward understanding the output of statistical learning. It revealed that infants detect and store generalizable representations of words and cross word sequences that can serve as object labels. However, the Switch task is limited in its ability to detect fine-grained differences in learners’ representations of novel word forms.

Advances in infant testing methodologies may help to address some of the limitations of these behavioral methods and present additional means of exploring questions about statistical word segmentation. Neurophysiological measures have potential to reveal characteristics of learning that may be masked by behavioral methodologies. Recent studies indicate that measures of brain activity, event-related potentials (ERPs), can provide more sensitive measures of infant word segmentation than listening time measures (Kooijman et al., 2005, 2009; Goyet et al., 2010). ERPs have also provided some evidence that newborns can track transitional probabilities in speech streams (Teinonen et al., 2009). Furthermore, Cunillera et al. (2006) recorded ERPs during statistical word segmentation in adults. They concluded that the timing of adults’ neural activity was consistent with the hypothesis that adults extract possible lexical units. Similar ERP evidence with infants would help to strengthen the claim that infants discover candidate words during statistical learning.

In conclusion, the results of the present experiments indicate that during statistical learning, infants form representations that are sufficiently abstract and flexible to recognize them across acoustic variation. Infants can perform this generalization to recognize words and to support other linguistic processes, in this case, associating the sounds of words with meanings. These findings suggest that statistical learning can withstand acoustic challenges present in infants’ language environments, which support the case for statistical learning as a viable contributor to language acquisition.

## Conflict of Interest Statement

The author declares that the research was conducted in the absence of any commercial or financial relationships that could be construed as a potential conflict of interest.
